# Family history of malignant tumor is a predictor of gastric cancer prognosis: Incorporation into a nomogram

**DOI:** 10.1097/MD.0000000000030141

**Published:** 2022-09-02

**Authors:** Fanke Wang, Liqiao Fan, Qun Zhao, Yu Liu, Zhidong Zhang, Dong Wang, Xuefeng Zhao, Yong Li, Bibo Tan

**Affiliations:** a Department of Gastrointestinal Surgery, The Fourth Hospital of Hebei Medical University, Shijiazhuang, P.R. China.

**Keywords:** family history of malignant tumor, gastric cancer, nomogram, overall survival, prediction model

## Abstract

The purpose of this study was to investigate the impact of a family history of malignant tumor on the prognosis of patients with gastric cancer and develop a nomogram that incorporates a family history of malignant tumor to predict overall survival (OS) in patients with gastric cancer to aid clinicians and patients in decision making. Four hundred eighty-eight patients with gastric cancer undergoing radical gastrectomy in our center were included and randomly split into a training set (n = 350) and a validation set (n = 138) at a ratio of 7:3. Cox univariate regression analysis was used to evaluate the influence of clinicopathological characteristics and family history of malignant tumors on their prognosis, and variables were screened by multivariate Cox regression analysis and consensus on clinical evidence. A nomogram was constructed for OS based on the filtered variables, and the C-index, receiver operating characteristic curve (ROC curve), and calibration curve were used to validate the nomogram and decision curve analysis curve (DCA curve) was used for clinical practicality assessment. Six variables related to OS, including the pathological differentiation degree, Lauren type, infiltration depth, lymph node metastasis, tumor deposit, and family history of malignant tumor, were screened to construct a nomogram. The nomogram developed in this study performed well in the training set and the validation set, with C-index of 0.776 and 0.757, and the area under the ROC curve(AUC) for predicting 1-, 3-, and 5-year survival rates are 0.838, 0.850, 0.820 and 0.754, 0.789, 0.808, respectively. The calibration curve shows that the estimated death risk of the nomogram in the 2 data sets is very close to the actual death risk. The net benefits of nomogram-guided prediction of patient survival at 1-, 3-, and 5 years were demonstrated by the DCA curves, which showed high clinical practicability. Family history of malignant tumors is an independent risk factor affecting the prognosis of patients with gastric cancer. The nomogram developed in this research can be used as an important tool to predict the prognosis of gastric cancer patients with family history data.

## 1. Introduction

Gastric cancer is a common malignant tumor of the digestive tract, and it has become the third leading cause of death in the world.^[1]^ Approximately 90% of gastric cancers worldwide are sporadic, while 10% of gastric cancers have family clusters.^[2]^ Family history of gastric cancer (FGC) is recognized as one of the high risk factors for the onset of gastric cancer.^[3–5]^ However, the relationship between the family history of other malignant tumors and the onset, clinicopathological characteristics, and prognosis of gastric cancer patients is still controversial.^[6,7]^ The identification of gastric cancer patients at high risk of mortality with a family history of tumors, and the early institution of aggressive therapeutic measures has certain clinical implications. Nomograms are an integrated model tool for medically and oncologically relevant prognostication and are typically used in the study of oncological outcomes compared to conventional staging, which enables the assessment of personalized risks through different patient circumstances.^[8]^

Therefore, this retrospective study explores the impact of family history of malignant tumors on the prognosis of gastric cancer patients, meanwhile, it was intended to establish a predictive model for predicting the 1 -, 3 -, and 5-year survival rates of patients with gastric cancer, while its performance and clinical usefulness were evaluated.

## 2. Materials and Methods

### 2.1. Research objects

**Figure 1.**
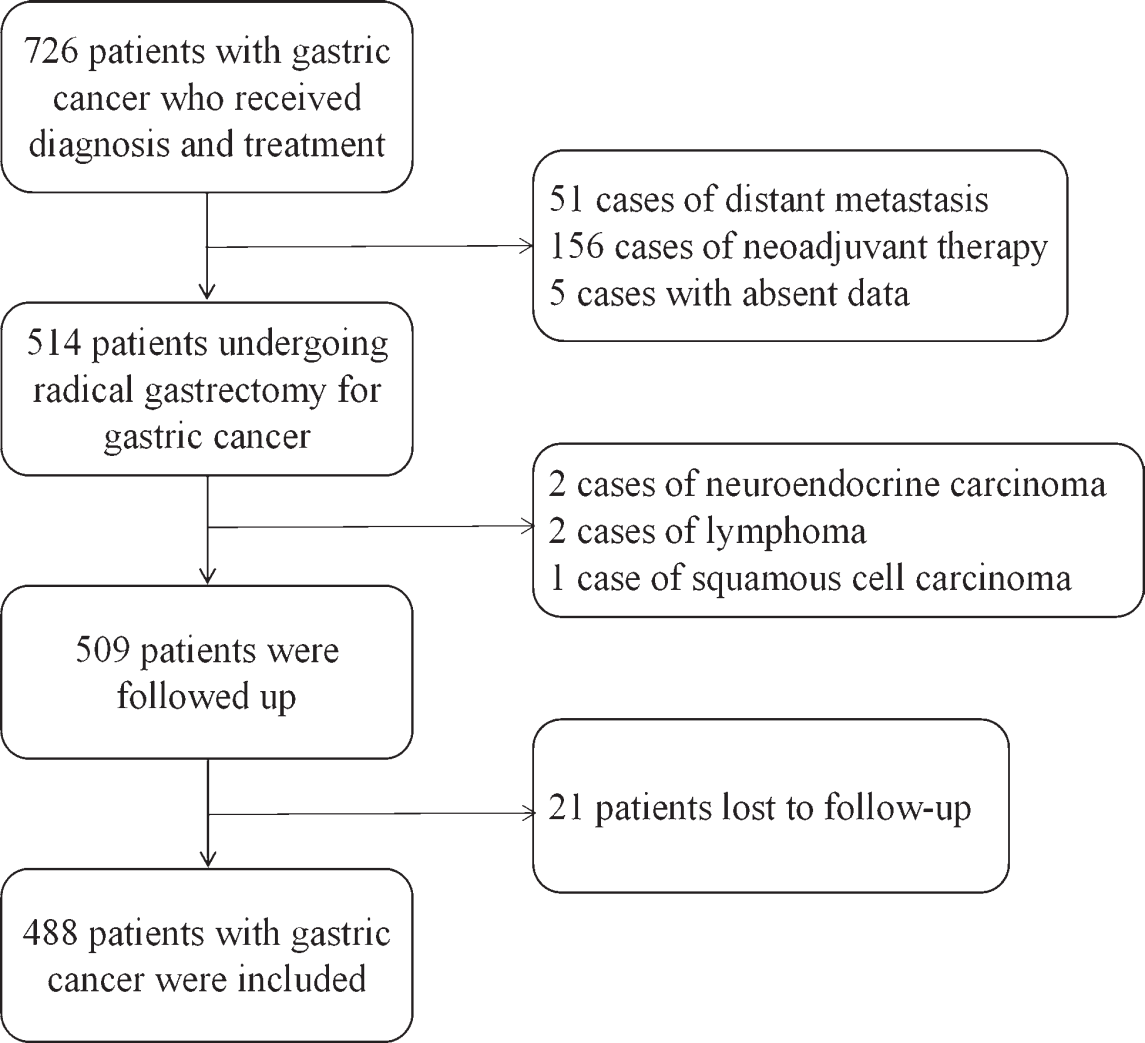
Screening flow diagram of patients.

Clinical data of all patients who underwent radical gastrectomy for gastric cancer in our center from January 1, 2016, to December 31, 2016, were collected, including age (at the time of diagnosis), gender, tumor length and diameter, degree of pathological differentiation, Lauren type, depth of invasion, level of lymph node metastasis, vascular tumor thrombus, nerve invasion, presence of tumor deposits, and family history of malignant tumor. Inclusion criteria: (1) Gastric adenocarcinoma diagnosed by histopathological examination with complete clinical pathological data; (2) radical gastrectomy (total gastrectomy or subtotal gastrectomy combined with lymph node dissection); (3) Systemic treatment is not received before operation; (4) Having complete and clear information about the family history of malignant tumors in the first-degree relatives and second-degree relatives; (5) Follow-up was obtained and follow-up data were complete. Exclusion criteria: (1) nonadenocarcinoma diagnosed by histopathological examination (including squamous cell carcinoma and neuroendocrine carcinoma); (2) The distant metastasis such as liver, lung, and abdominal cavity is confirmed; (3) Palliative surgery, simple biopsy or endoscopic treatment is performed; (4) Other tumors are combined (Figure 1). This study used the minimum standards for family history of malignant tumors established by the American Society of Clinical Oncology in 2014.^[9]^ Among the patients’ first-degree relatives and second-degree relatives, there are patients who are pathologically diagnosed as malignant tumors. First-degree relatives include parents, children, and siblings. Second-degree relatives include grandparents, uncles and aunts, nephews and nieces, and grandchildren. Family history of gastric cancer is defined as patients whose first-degree relatives and/or second-degree relatives are pathologically diagnosed as gastric adenocarcinoma. Family history of other malignant tumor is defined as patients whose first-degree relatives and/or second-degree relatives have pathologically confirmed other malignant tumors, but no patients with gastric adenocarcinoma. No family history of malignant tumor is defined as patients whose first-degree and second-degree relatives have no confirmed malignant tumor. Finally, a total of 488 cases that met the criteria were included.

### 2.2. Follow-up data

Patients were followed up for information such as survival state, survival time, and cause of death. The follow-up deadline was December 31, 2021. OS is defined as the period from the date of diagnosis of gastric cancer to the date of death (from any cause) or the date of last follow-up. Collection and evaluation of the patient’s family history of malignant tumors at the initial visit, including information on the primary malignancy of the primary and secondary relatives, consanguinity to the patient, age of diagnosis,^[10]^ family history was reassessed and updated at each subsequent visit, and the study was conducted using updated family history information from the last visit. The information obtained in this study does not involve patient privacy.

### 2.3. Statistical analysis

The present data set was randomly split in a 7:3 ratio into a training set (n = 350) and a verification set (n = 138) using the R function “createDataPartition.”^[11]^ Factors associated with OS were screened using univariate Cox regression analysis in the training set. Variables that were statistically significant in the univariate Cox analysis were subjected to Cox multivariate regression analysis to screen for independent risk factors, and the regression was conducted step by step. The Akaike information criterion was used to measure the superiority of the model, and the variables were screened based on the clinical evidence and consensus. The selected variables followed the Harrell principle.^[8]^ Finally, based on the screened influencing factors, the nomograms for predicting the 1-, 3-, and 5-year survival rates of patients with gastric cancer were constructed using the packages “rms,” “survival,” “riskRegression,” etc, in the training group, C-index, ROC curve, and calibration curve were used to verify the nomograms, and DCA curve was used to evaluate clinical applicability.^[12]^ The scores of all patients in the validation set were calculated by the established prediction model to validate the validation set data with the scores. All analyses were performed using version 4.1.2 of the R software. *P* < .05 indicated that the difference was statistically significant.

## 3. Results

### 3.1. Clinical pathological data and univariate COX analysis

A total of 488 patients with gastric cancer were included, 96 with a family history of malignant tumors in first- and second-degree relatives, 42 with a family history of gastric cancer, 54 with a family history of other malignant tumors, 350 in the training set and 138 in the validation set. All cases were diagnosed at the age of 15 to 80 years old, and the median follow-up time was 60.12 (0.7–72.6) months. The overall 1-, 3-, and 5-year survival rates were 89.75%, 69.88%, and 63.73%, respectively (Table 1). Univariate COX analysis showed that tumor length, pathological differentiation, Lauren type, depth of invasion, level of lymph node metastasis, vascular tumor thrombus, nerve invasion, presence of tumor deposit, and family history of malignant tumors were the relevant factors for OS in patients (*P* < .05) (Table 2).

**Table 1:** Clinical and pathological data of gastric cancer patients.

Characteristics	Total (n = 488)		Training set (n = 350)		Validation set (n = 138)	
	Cases	Ratio (%)	Cases	Ratio (%)	Cases	Ratio (%)
Age (y)						
≥50	424	86.9	311	88.9	113	81.9
<50	64	13.1	39	11.1	25	18.1
Sex						
Male	369	75.6	264	75.4	105	76.1
Female	119	24.4	86	24.6	33	23.9
Tumor maximum diameter*						
≥4 cm	266	54.5	192	54.9	74	53.6
<4 cm	222	45.5	158	45.1	64	46.4
Differentiation degree†						
Low	237	48.6	163	46.6	74	53.6
Medium-high	251	51.4	187	53.4	64	46.4
Lauren type						
Intestinal	144	29.5	108	30.9	36	26.1
Diffuse	228	46.7	159	45.4	69	50.0
Mixed	116	23.8	83	23.7	33	23.9
T stage‡						
T1	99	20.3	75	21.4	24	17.4
T2	68	13.9	47	13.4	21	15.2
T3	12	2.5	9	2.6	3	2.2
T4a	299	61.3	214	61.1	85	61.6
T4b	10	2.0	5	1.4	5	3.6
N stage‡						
0	197	40.4	140	40.0	57	41.3
1	78	16.0	51	14.6	27	19.6
2	91	18.6	67	19.1	24	17.4
3a	67	13.7	52	14.9	15	10.9
3b	55	11.3	40	11.4	15	10.9
Vascular tumor thrombus						
Yes	161	33.0	119	34.0	42	30.4
No	327	67.0	231	66.0	96	69.6
Nerve invasion						
Yes	256	52.5	183	52.3	73	52.9
No	232	47.5	167	47.7	65	47.1
Tumor deposit						
Yes	44	9.0	30	8.6	14	10.1
No	444	91.0	320	91.4	124	89.9
Family history of malignant tumor						
No	392	80.3	284	81.1	108	78.3
Gastric	42	8.6	28	8.0	14	10.1
Other	54	11.1	38	10.9	16	11.6

* Median maximum diameter.

† World Health Organization(WHO) pathological type of gastric cancer: medium-high differentiation includes papillary adenocarcinoma and tubular adenocarcinoma, and low differentiation includes poorly differentiated adenocarcinoma, mucinous adenocarcinoma, and signet-ring cell carcinoma.

‡ American Joint Committee on Cancer(AJCC) 8th edition TNM staging of gastric cancer.

**Table 2:** univariate COX analysis of gastric cancer patients in training set.

Characteristics	HR (95% CI)	*P*
Age(y)		
≥50	Reference	
<50	0.69 (0.42–1.14)	.15
Sex		
Male	Reference	
Female	0.89 (0.59–1.34)	.582
Tumor maximum diameter		
≥4 cm	Reference	
<4 cm	2.76 (1.89–4.03)	<.001
Differentiation degree		
Low	Reference	
Medium-high	0.52 (0.37–0.74)	<.001
Lauren type		
Intestinal	Reference	
Diffuse	3.96 (2.4–6.53)	<.001
Mixed	2.63 (1.49–4.62)	.001
T stage		
T1	Reference	
T2	1.35 (0.45–4.01)	.593
T3	7.37 (2.34–23.24)	.001
T4a	7.62 (3.55–16.37)	<.001
T4b	20.82 (6.07–71.38)	<.001
N stage		
0	Reference	
1	2.22 (1.17–4.23)	.015
2	3.21 (1.84–5.58)	<.001
3a	7.02 (4.09–12.03)	<.001
3b	11.1 (6.44–19.16)	<.001
Vascular tumor thrombus		
Yes	Reference	
No	2.34 (1.66–3.28)	<.001
Nerve invasion		
Yes	Reference	
No	3.21 (2.19–4.7)	<.001
Tumor deposit		
Yes	Reference	
No	2.77 (1.75–4.39)	<.001
Family history of malignant tumor		
No	Reference	
Gastric cancer	1.89 (1.1–3.25)	.021
Other	1.08 (0.63–1.86)	.771

### 3.2. Multivariate COX regression analysis

**Figure 2.**
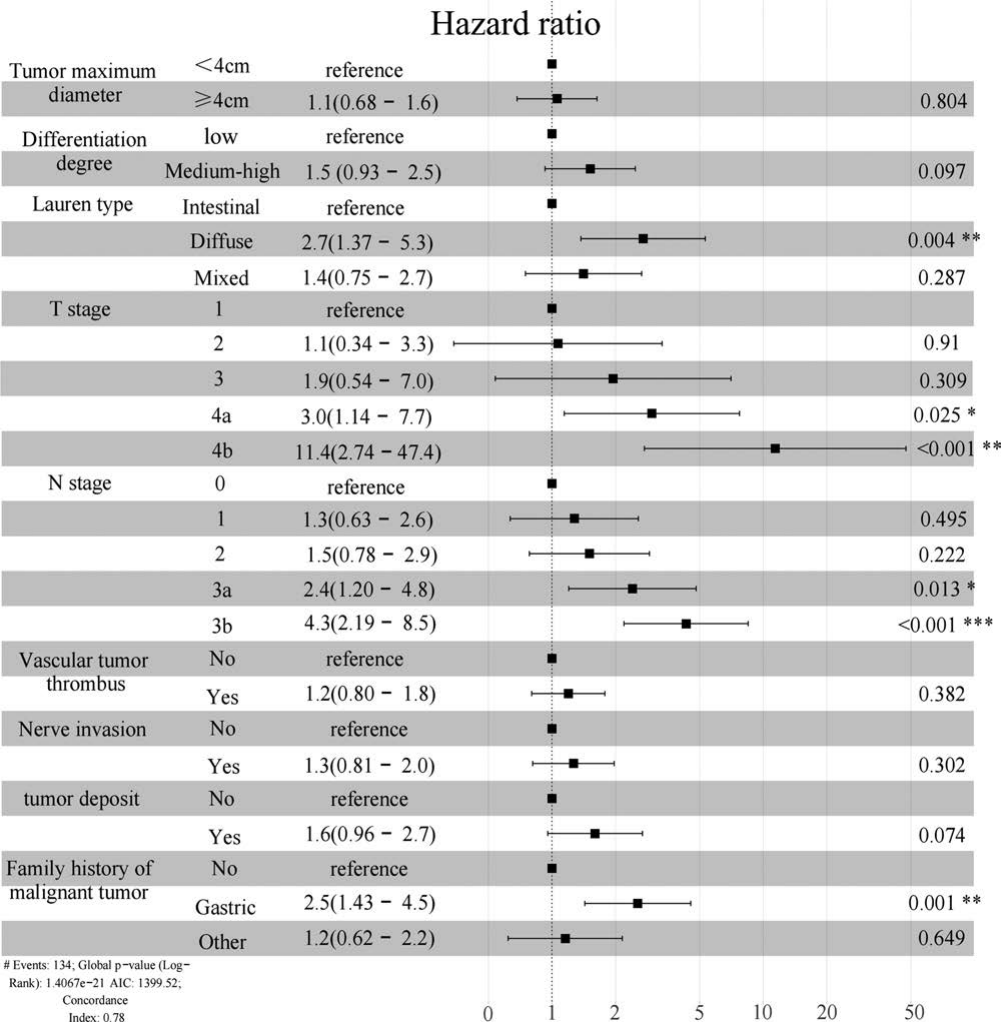
HR, 95% CI, and forest plots for OS multivariate Cox regression analysis by training set. CI = confidence intervals, HR = hazard ratio, OS = overall survival.

Statistical significances of univariate COX analysis were incorporated into multivariate COX regression analysis, and stepwise regression was conducted backwards. Screening according to Akaike information criterion criteria showed that the independent prognostic risk factors for gastric cancer were Lauren type, depth of invasion, lymph node metastasis, and family history of malignant tumor (*P* < .05). However, the degree of differentiation (*P* = .097) and the presence of tumor deposits (*P* = .074) were marginally significant (Fig. 2).

### 3.3. Construction of nomogram

**Figure 3.**
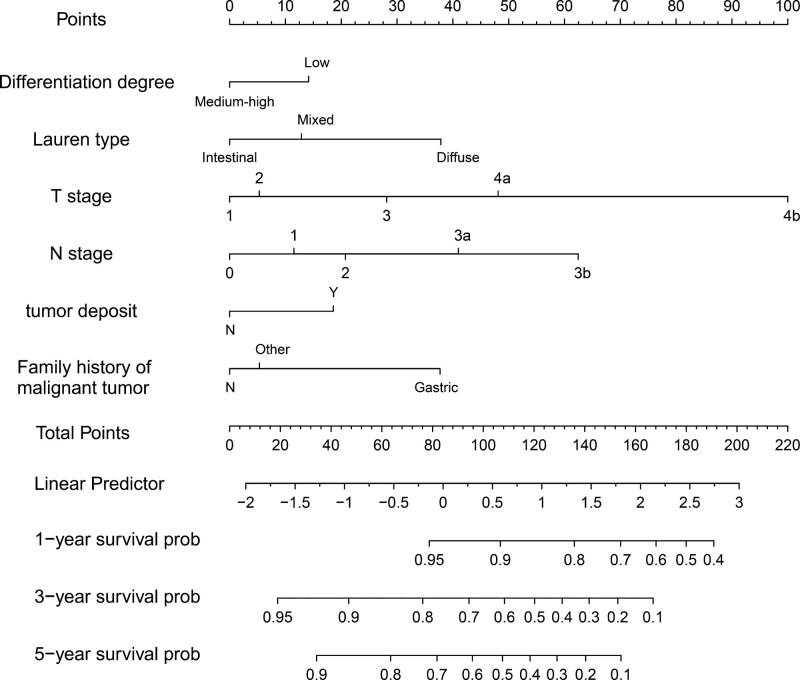
1-, 3-, and 5-year OS nomogram for patients with gastric cancer. OS = overall survival.

In order to better predict the results, we screened 4 independent factors (Lauren classification, invasion depth, lymph node metastasis, and family history of malignant tumors) and 2 related factors (differentiation degree and presence of tumor deposit) to construct nomogram (Fig. 3). The long diameter of tumor, vascular tumor thrombus and nerve invasion were eliminated in the variable screening process. In the nomogram, the total score is obtained by summing the scores corresponding to each factor. The scale at the bottom of the total score indicates the 1-, 3-, and 5-year survival rates.

### 3.4. Validation of nomogram

**Figure 4.**
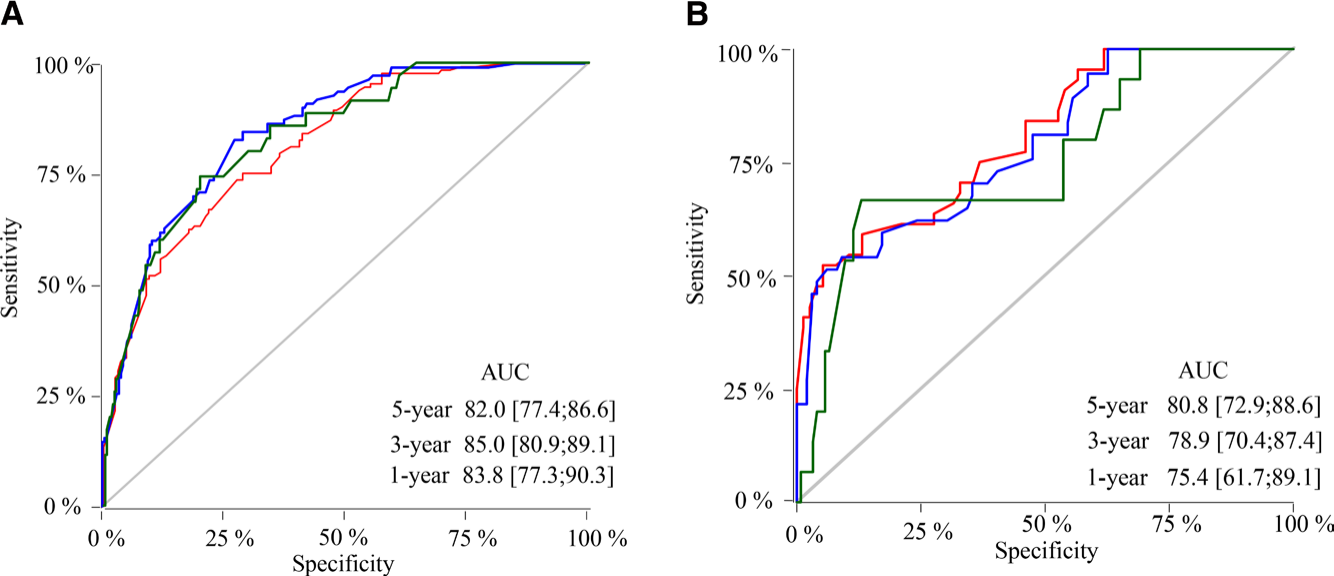
ROC curves for 1-, 3-, and 5-year survival for the training set and the validation set. (A) Training set; (B) validation set. ROC = receiver operating characteristic.

**Figure 5.**
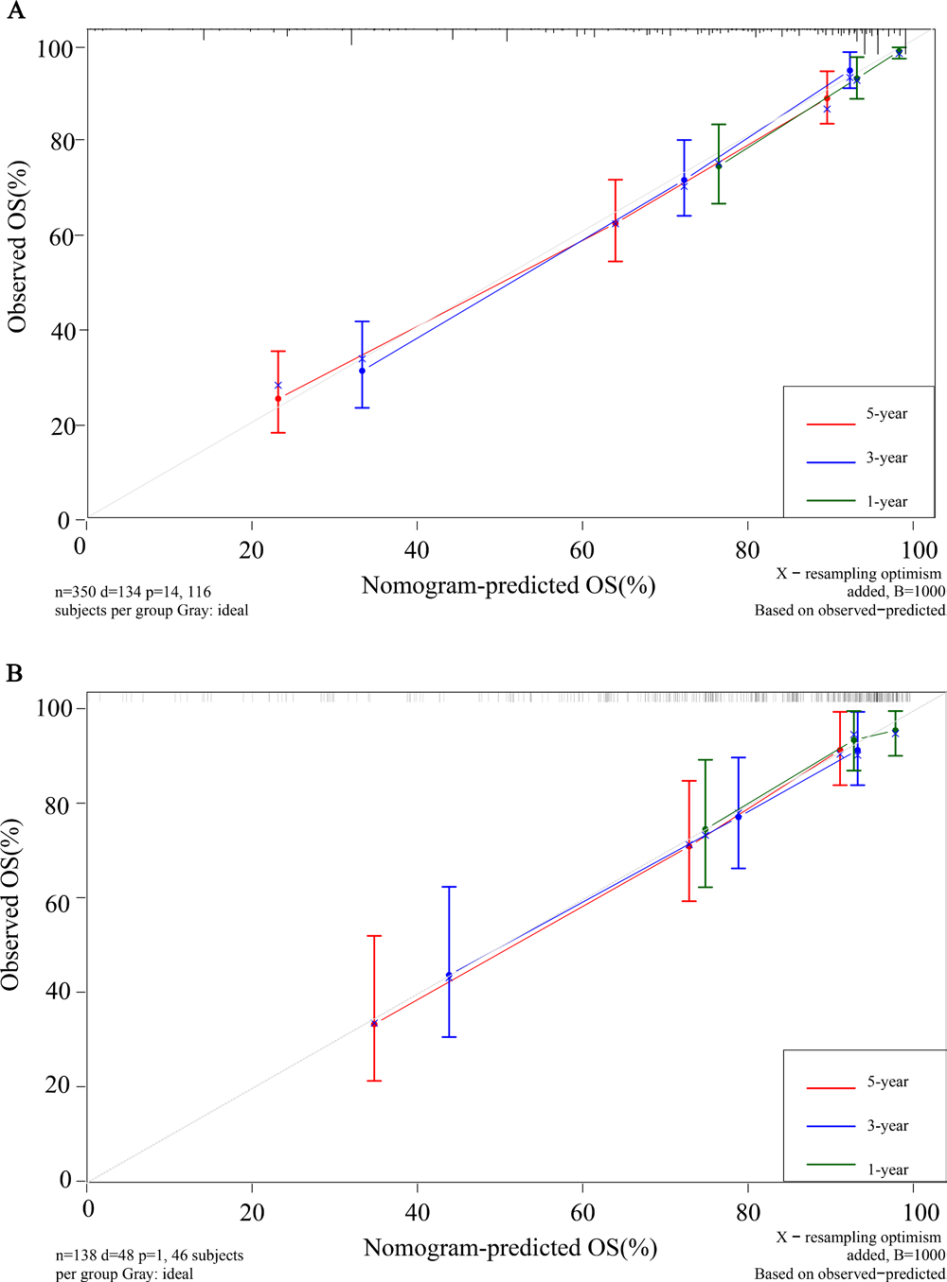
Calibration curves for the training set and the validation set. (A) training set; (B) validation set.

Through the established prediction model, the scores of all patients in the validation set were calculated, and the data in the validation set were verified with the score as the unique new variable. The nomogram constructed by the training set predicted a C-index of 0.776 and the validation set was 0.757. The areas under the ROC curve for 1-, 3-, and 5-year survival in the training set were 0.838 (95% CI: 77.3–90.3), 0.850 (95% CI: 80.9–89.1), and 0.820 (95% CI: 77.4–86.6), respectively; 0.754 (95% CI: 61.7–89.1), 0.789 (95% CI: 70.4–87.4), and 0.808 (95% CI: 72.9–88.6) in the validation group, respectively (Fig. 4). Calibration curves show that the nomograms constructed in this study predicted very close 1-, 3-, and 5-year survival to actual survival (Fig. 5).

### 3.5. Clinical practicability

**Figure 6.**
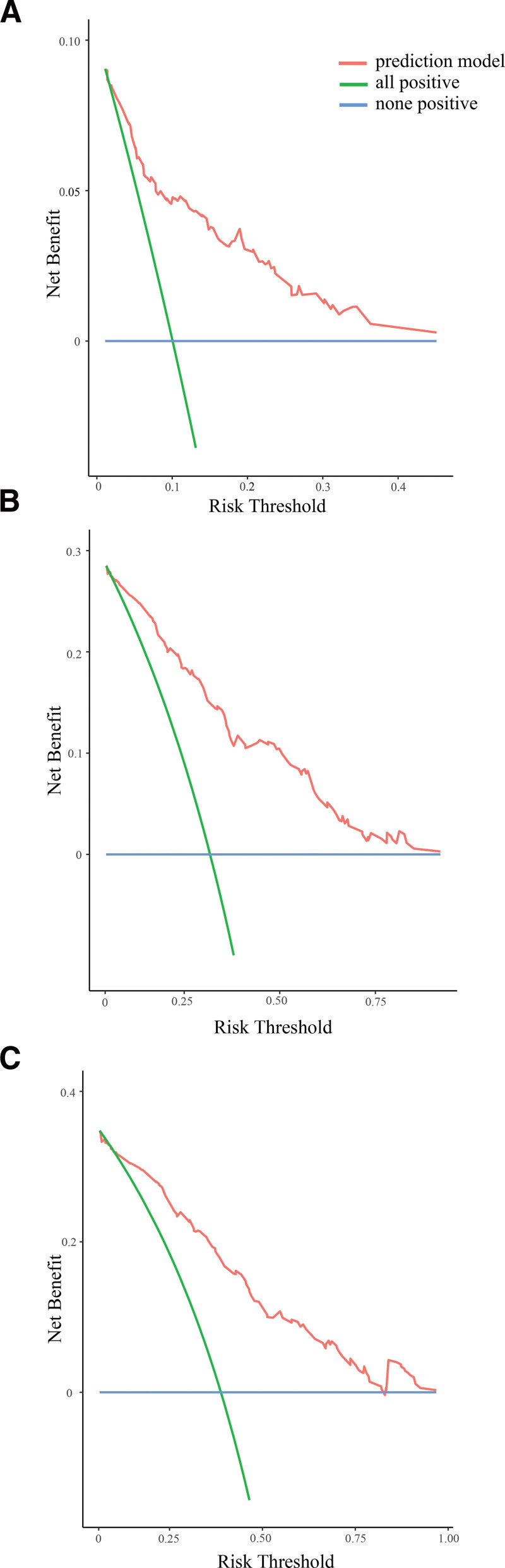
1-, 3-, and 5-year DCA curves of nomogram. (A) 1-year DCA curve; (B) 3-year DCA curve; (C) 5-year DCA curve. DCA = decision curve analysis.

Clinical practicability is used to assess whether nomogram-aided decisions improve patient outcomes. The net benefit (y) of the blue line is 0. Under the same threshold probability (x), the net benefit of the red line is higher than that of the green line. The nomogram-guided 1-, 3-, and 5-year predictions of net gains in patient survival were both high and clinically useful (Fig. 6).

## 4. Discussion

The genetic study of gastric cancer lags behind other gastrointestinal tumors, such as colorectal cancer, and multiple susceptibility genes have been reported.^[13,14]^ Different from colorectal cancer, gastric cancer is more common in low-income countries, where the population’s education and concept of life affect epidemiological research such as biological sample collection or census, follow-up, and basic research and clinical practice of cancer genetics are limited by funding and the scarcity of medical professionals in this field.^[15]^ At present, some studies have reported the relationship between the family history of cancer in the first-degree relatives and/or the second-degree relatives and the risk, clinical, and pathological characteristics and prognosis of gastric cancer. Some studies have shown that the family history of malignant tumors is related to the poor prognosis of gastric cancer patients.^[7,16]^ However, some studies have also shown that the family history of malignant tumors is not related to the prognosis of gastric cancer patients.^[17,18]^ At the same time, patients with malignant tumors that already exist in the family may improve the health awareness of other members of the family, so that some gastric cancers can be found early in physical examination.^[19]^ Some studies show that the prognosis of gastric cancer patients with family history of malignant tumors is better than those without family history.^[6,20,21]^ We collected the clinicopathological factors that were not involved in most studies, including tumor family history, tumor deposit, Lauren classification, but might affect the prognosis. At the first visit, we evaluated the family history of malignant tumors of the first and second relatives of patients for the first time, and updated the content of the family history of tumors in each follow-up, and classified the family history into the family history of gastric cancer and other malignant tumors according to the types of relatives suffering from malignant tumors. Multivariate COX regression analysis showed that the family history of malignant tumors, especially the family history of gastric cancer, was an independent risk factor affecting the prognosis of patients with gastric cancer (OS: HR = 2.5,95% CI: 1.43–4.5).

Compared with previous prediction models for the prognosis of gastric cancer,^[22,23]^ for the first time, family history of tumor was included in the nomogram as a factor affecting prognosis. Nomograms can simplify the complex prediction model into an estimation of the probability of occurrence of events (such as death, recurrence, progression, and so on) according to the clinical and pathological conditions of each patient; nomograms were constructed and validated by selection of statistically or clinically significant variables.^[24]^ Univariate COX analysis showed that gender and age had nothing to do with the prognosis of gastric cancer, while factors such as the longest diameter of tumor, vascular tumor thrombus, and nerve invasion might be related to the prognosis. However, multivariate COX analysis showed that their influence was not enough to be included in the prediction model, so they were eliminated in the further screening of variables. In addition, the cutoff values (4 cm, 5 cm, 8 cm, 10 cm) were different in previous studies on the longest diameter of tumor and the prognosis of gastric cancer.^[25,26]^ In this study, the median longest diameter (4 cm) of the data was used as the cutoff value.

Lauren classification is classified according to the histological structure and biological behavior of gastric cancer, with high consistency among different observers, and is a commonly used histological classification standard for gastric cancer.^[27]^ Some studies have shown that compared with intestinal type of gastric cancer, diffuse type has a poor prognosis, and Lauren classification can be used as a marker of prognosis for patients with gastric cancer after radical resection.^[28–30]^ This study also shows that Lauren type is an independent risk factor for prognosis of gastric cancer. Tumor deposit, also known as soft tissue extranodal metastases, are defined as the presence of tumor cells in the extramural soft tissue of the stomach that is not contiguous with either the tumor primary or the regional lymph nodes. Previous studies have shown that tumor deposit portend worse prognosis for gastric cancer.^[31–33]^ For patients after radical gastrectomy for gastric cancer, tumor deposit are an independent risk factor for prognosis, and their significance for prognosis is different from lymph node metastasis of gastric cancer. Some scholars believe that they should be classified separately from lymph node metastasis.^[34]^ In this study, the tumor deposit was included in the model as a separate variable that could be used as a complement to traditional TNM stage. The degree of differentiation of gastric cancer also has important clinical significance for prognosis.^[35]^ Moreover, multivariate analysis in this study showed that the tumor deposit and the degree of differentiation were marginally significant (*P* < .1). the nomograms constructed using these variables performed well in both the training set and the verification set without overfitting.^[36]^ Therefore, 6 variables are finally selected to construct the nomogram.

External validation using the validation data set after the completion of nomogram construction showed no significant differences in the C-index or the area under the ROC curve between the training and validation sets, and the calibration curves all showed that the predicted survival was very close to the actual survival. The DCA curve showed a higher net benefit for patients guided by the nomogram.

Despite the good performance of the nomogram, this study has certain limitations. First, this study was a retrospective case study, which will inevitably generate selection bias, meanwhile the single center, small sample size of the study subjects limited the applicability of the results; Second, the study did not include the status of postoperative adjuvant therapy, patients’ nutritional status, and blood tumor marker results, which may affect the prognosis. Some results still need to be confirmed by large sample, multicenter prospective randomized controlled trials, but it is undeniable that this study is the first to incorporate family history of malignant tumor as a factor of prognosis in gastric cancer patients into a nomogram, and all predictor variables are easily accessible clinically, which can be used as an important tool for assessing survival in gastric cancer patients with family history and has certain clinical value.

## 5. Conclusion

The family history of malignant tumor may be an adverse factor affecting the prognosis of patients with gastric cancer. The nomogram constructed in this study can predict the survival rate of gastric cancer patients with family history of malignant tumors and will be helpful for clinicians and patients with gastric cancer to make decisions.

## Acknowledgments

We appreciate all participants in this work.

## Author contributions

Conceptualization: Yong Li, Bibo Tan

Data acquisition: Fanke Wang, Yu Liu, Zhidong Zhang, Dong Wang, Xuefeng Zhao

Data analysis: Liqiao Fan, Qun Zhao

Interpretation: Liqiao Fan, Qun Zhao

Writing – original draft:Fanke Wang

Writing – review & editing:Fanke Wang, Yong Li, Bibo Tan
